# [*meso*-5,10,15,20-Tetra­kis(3-methyl­thio­phen-2-yl)porphyrinato-κ^4^ *N*,*N*′,*N*′′,*N*′′′]nickel(II) benzene hemisolvate

**DOI:** 10.1107/S1600536813030468

**Published:** 2013-11-13

**Authors:** R. Prasath, P. Bhavana, Sushil K. Gupta, Ray J. Butcher

**Affiliations:** a Department of Chemistry, BITS, Pilani – K. K. Birla Goa Campus, Goa 403 726, India; b School of Studies in Chemistry, Jiwaji University, Gwalior 474 011, India; c Department of Chemistry, Howard University, 525 College Street NW, Washington, DC 20059, USA

## Abstract

In the title compound, [Ni(C_40_H_28_N_4_S_4_)]·0.5C_6_H_6_, the Ni^II^ atom is in a square-planar geometry defined by four pyrrole N atoms. There is considerable buckling in the porphyrin ring with the dihedral angles between the N_4_ donor set and the pyrrole rings being in the range 16.24 (5)–22.47 (5)°. Each of the six-membered chelate rings is twisted about an Ni—N bond and the dihedral angles between diagonally opposite chelate rings are 21.36 (4) and 23.87 (4)°; each pair of rings is oriented in opposite directions. The methyl­thienyl rings are twisted out of the plane of the central N_4_ core with dihedral angles in the range 75.98 (2)–88.70 (5)°. All four methyl­thienyl groups are disordered over two sets of sites, as is commonly found with such groups, with occupancies of 0.553 (8):0.447 (8), 0.579 (7):0.421 (7), 0.796 (6):0.204 (6) and 0.956 (7):0.044 (7). The benzene solvent mol­ecule was found to be present in half-occupancy.

## Related literature

For related structures, see: Prasath *et al.* (2012*a* ▶,*b* ▶); Purushothaman *et al.* (2001 ▶); Song *et al.* (2005 ▶). For the synthesis, see: Sun *et al.* (2005 ▶); Prasath *et al.* (2012*a* ▶). For general background and potential applications of thienylporphyrins, see: Boyle *et al.* (2010 ▶); Rochford *et al.* (2008 ▶); Chen *et al.* (2010 ▶); Friedlein *et al.* (2005 ▶); Wallin *et al.* (2006 ▶).
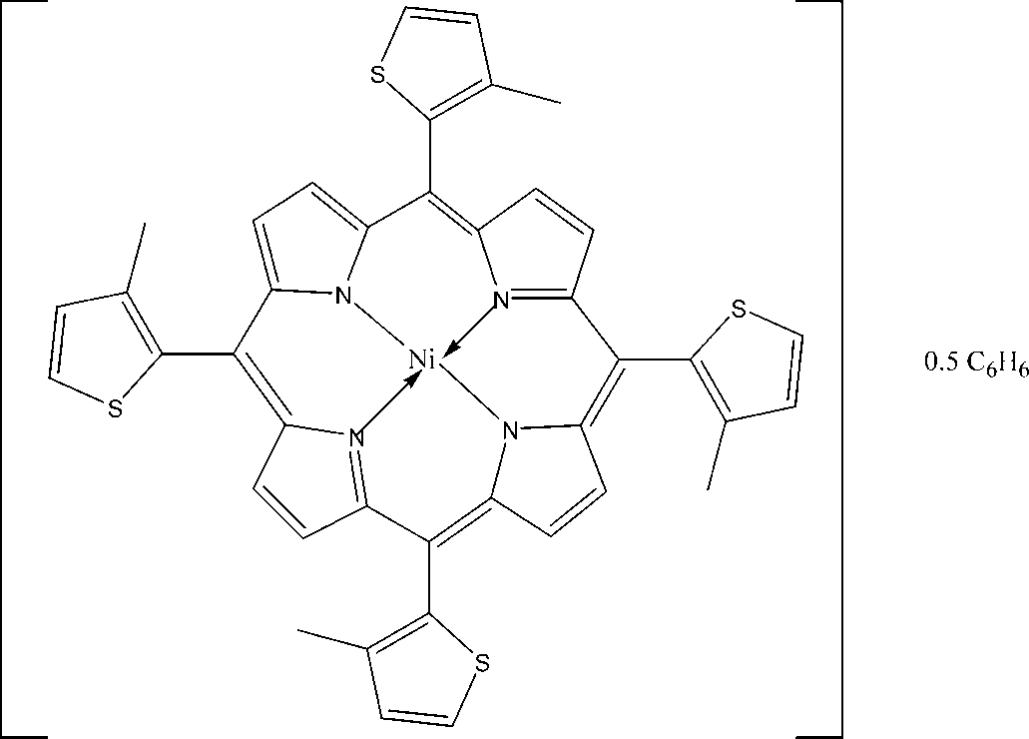


## Experimental

### 

#### Crystal data

[Ni(C_40_H_28_N_4_S_4_)]·0.5C_6_H_6_
*M* *_r_* = 790.67
Orthorhombic, 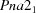

*a* = 12.4854 (6) Å
*b* = 11.3906 (5) Å
*c* = 28.365 (2) Å
*V* = 4034.0 (4) Å^3^
*Z* = 4
Mo *K*α radiation
μ = 0.72 mm^−1^
*T* = 295 K
0.39 × 0.22 × 0.05 mm

#### Data collection

Agilent Xcalibur (Ruby, Gemini) diffractometer
Absorption correction: analytical [*CrysAlis PRO* (Agilent, 2012 ▶), using a multi-faceted crystal model (Clark & Reid (1995 ▶)] *T*_min_ = 0.830, *T*_max_ = 0.965
12922 measured reflections
5674 independent reflections
2720 reflections with *I* > 2σ(*I*)
*R*_int_ = 0.091

#### Refinement

*R*[*F* ^2^ > 2σ(*F* ^2^)] = 0.077
*wR*(*F* ^2^) = 0.240
*S* = 0.99
5674 reflections
562 parameters
709 restraints
H-atom parameters constrained
Δρ_max_ = 0.32 e Å^−3^
Δρ_min_ = −0.24 e Å^−3^
Absolute structure: Flack (1983 ▶), 55% of Friedels measured
Absolute structure parameter: 0.35 (4)

### 

Data collection: *CrysAlis PRO* (Agilent, 2012 ▶); cell refinement: *CrysAlis PRO*; data reduction: *CrysAlis PRO*; program(s) used to solve structure: *SHELXS97* (Sheldrick, 2008 ▶); program(s) used to refine structure: *SHELXL97* (Sheldrick, 2008 ▶); molecular graphics: *SHELXTL* (Sheldrick, 2008 ▶); software used to prepare material for publication: *SHELXTL*.

## Supplementary Material

Crystal structure: contains datablock(s) I. DOI: 10.1107/S1600536813030468/zs2280sup1.cif

Structure factors: contains datablock(s) I. DOI: 10.1107/S1600536813030468/zs2280Isup2.hkl

Additional supplementary materials:  crystallographic information; 3D view; checkCIF report

## Figures and Tables

**Fig. 1.**
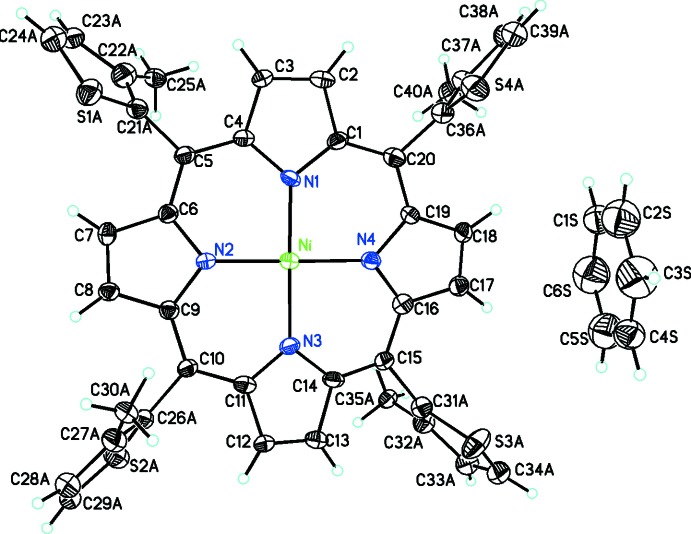
Diagram of the title compound showing the atom labeling scheme and 30% probability displacement ellipsoids showing the major component only, and including the benzene solvate molecule.

**Fig. 2.**
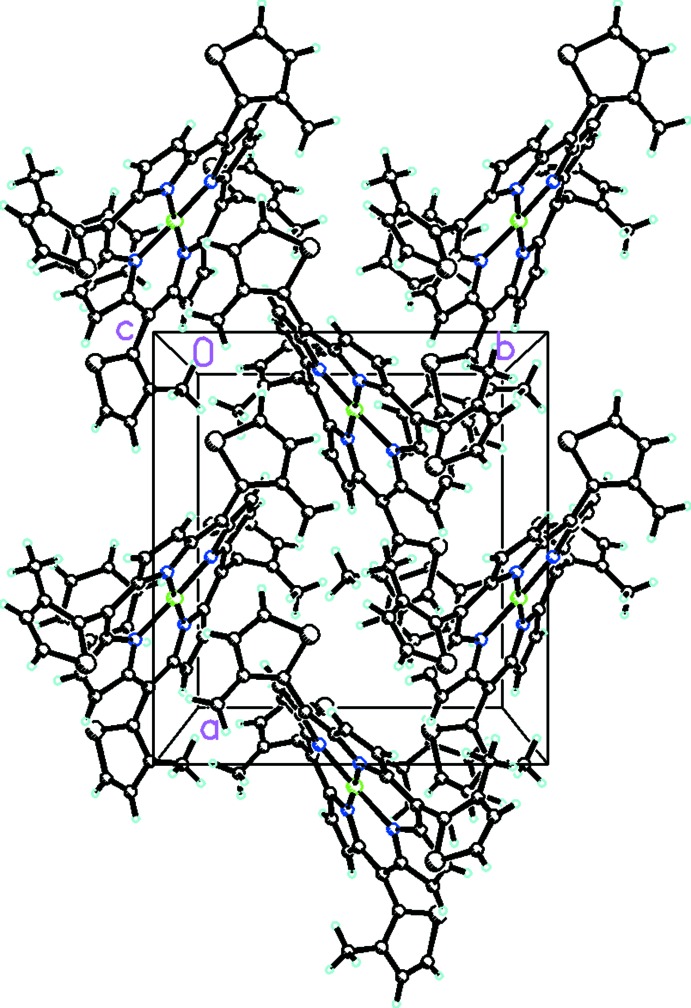
Molecular packing of Ni(C40H28N4S4)·0.5(C6H6) viewed along the c axis.

## supplementary crystallographic information

### 1. Comment

Thienylporphyrins are of growing interest owing to physiochemical (Boyle *et al.*, 2010), energy transfer (Wallin *et al.*, 2006), thin films (Friedlein *et al.*,2005), electrochemical (Chen *et al.*, 2010) and photophysical (Rochford *et al.*, 2008) properties. In continuation of our work on thienylpophyrins (Prasath *et al.*, 2012*a*, 2012*b*; Purushothaman *et al.*, 2001), we report herein the crystal structure of 5,10,15,20-tetrakis(3-methylthien-2-yl)porphyrinatonickel(II) hemi(benzene) solvate (Fig.1). The 24-membered porphyrin moiety of the title compound is planar with a mean deviation of 0.0389 (3) Å. The Ni^II^ atom (Fig.1) is in a square planar geometry defined by four pyrrole-N atoms, Table 1. These bond lengths are in agreement with those found in other nickel porphyrin compounds (Prasath *et al.*, 2012*a*,*b*; Purushothaman *et al.*, 2001; Song *et al.*, 2005). There is considerable buckling in the porphyrin ring with the dihedral angles between the N_4_ donor set and the N1—N4 pyrrole rings of 16.24 (5), 20.87 (5), 20.52 (4) and 22.47 (5)°, respectively. Each of the six–membered chelate rings is twisted about an Ni–N bond and the dihedral angles between diagonally opposite chelate rings are 21.36 (4) and 23.87 (4)°; each pair of rings is oriented in opposite directions. The usual ruffling in the structure is further indicated by the deviations of C5, C15, C25 and C35 from the NiN_4_ plane [C5 (-0.4076 Å), C15 (0.4171 Å), C25 (-0.3973 Å), C35 (0.3878 Å)]. The methylthienyl rings are twisted out of the plne of the central N_4_ core with dihedral angles being 75.98 (2), 88.70 (5), 84.60 (5) and 85.44 (4)°, respectively. All four methylthienyl groups are disordered over two sets of sites, as is commonly found with such groups, with occupancies of 0.553 (8), 0.447 (8); 0.579 (7), 0.421 (7); 0.796 (6), 0.204 (6); and 0.956 (7), 0.044 (7), respectively. The benzene solvent molecule was found to be present in half occupancy.

### 2. Experimental

[*meso*-5,10,15,20-Tetrakis(3-methylthien-2-yl)porphyrinato- κ^4^*N*,*N*',*N*'',*N*''']nickel(II) hemi(benzene) solvate was synthesized as reported in the literature (Sun *et al.*, 2005; Prasath *et al.*, 2012*a*). Recrystallization by slow evaporation of a chloroform/benzene solution yielded purple crystals. Yield: 80%.

### 3. Refinement

All H atoms were placed in calculated positions and refined using a riding-model approximation with atom—H lengths of 0.93 Å (CH) or 0.96 Å (CH_3_). Isotropic displacement parameters for these atoms were set to 1.2 (CH) or 1.5 (CH_3_) times *U*_eq_ of the parent atom. All four methylthienyl groups are disordered over two sets of sites, as is commonly found with such groups, with occupancies of 0.553 (8), 0.447 (8); 0.579 (7), 0.421 (7); 0.796 (6), 0.204 (6); and 0.956 (7), 0.044 (7), respectively. The benzene solvent molecule has found to be present in half occupancy and was constrained to be hexagonal.

### Crystal data

**Table:**

[Ni(C_40_H_28_N_4_S_4_)]·0.5C_6_H_6_	*F*(000) = 1636
*M**_r_* = 790.67	*D*_x_ = 1.302 Mg m^−^^3^
Orthorhombic, *P**n**a*2_1_	Mo *K*α radiation, λ = 0.71069 Å
Hall symbol: P 2c -2n	Cell parameters from 1901 reflections
*a* = 12.4854 (6) Å	θ = 3.2–28.5°
*b* = 11.3906 (5) Å	µ = 0.72 mm^−^^1^
*c* = 28.365 (2) Å	*T* = 295 K
*V* = 4034.0 (4) Å^3^	Prism, dark purple
*Z* = 4	0.39 × 0.22 × 0.05 mm

### Data collection

**Table:**

Agilent Xcalibur (Ruby, Gemini) diffractometer	5674 independent reflections
Radiation source: Enhance (Mo) X-ray Source	2720 reflections with *I* > 2σ(*I*)
Graphite monochromator	*R*_int_ = 0.091
Detector resolution: 10.5081 pixels mm^-1^	θ_max_ = 25.3°, θ_min_ = 3.2°
ω scans	*h* = −14→14
Absorption correction: analytical [*CrysAlis PRO* (Agilent, 2012), using a multi-faceted crystal model (Clark & Reid (1995)]	*k* = −13→12
*T*_min_ = 0.830, *T*_max_ = 0.965	*l* = −20→34
12922 measured reflections	

### Refinement

**Table:**

Refinement on *F*^2^	Secondary atom site location: difference Fourier map
Least-squares matrix: full	Hydrogen site location: inferred from neighbouring sites
*R*[*F*^2^ > 2σ(*F*^2^)] = 0.077	H-atom parameters constrained
*wR*(*F*^2^) = 0.240	*w* = 1/[σ^2^(*F*_o_^2^) + (0.1154*P*)^2^] where *P* = (*F*_o_^2^ + 2*F*_c_^2^)/3
*S* = 0.99	(Δ/σ)_max_ = 0.005
5674 reflections	Δρ_max_ = 0.32 e Å^−^^3^
562 parameters	Δρ_min_ = −0.24 e Å^−^^3^
709 restraints	Absolute structure: Flack (1983), ???? Friedel pairs
Primary atom site location: structure-invariant direct methods	Absolute structure parameter: 0.35 (4)

### Special details

**Table:**

Geometry. All e.s.d.'s (except the e.s.d. in the dihedral angle between two l.s. planes) are estimated using the full covariance matrix. The cell e.s.d.'s are taken into account individually in the estimation of e.s.d.'s in distances, angles and torsion angles; correlations between e.s.d.'s in cell parameters are only used when they are defined by crystal symmetry. An approximate (isotropic) treatment of cell e.s.d.'s is used for estimating e.s.d.'s involving l.s. planes.
Refinement. Refinement of *F*^2^ against ALL reflections. The weighted *R*-factor *wR* and goodness of fit *S* are based on *F*^2^, conventional *R*-factors *R* are based on *F*, with *F* set to zero for negative *F*^2^. The threshold expression of *F*^2^ > σ(*F*^2^) is used only for calculating *R*-factors(gt) *etc*. and is not relevant to the choice of reflections for refinement. *R*-factors based on *F*^2^ are statistically about twice as large as those based on *F*, and *R*- factors based on ALL data will be even larger.

### Fractional atomic coordinates and isotropic or equivalent isotropic displacement parameters (Å^2^)

**Table:**

	*x*	*y*	*z*	*U*_iso_*/*U*_eq_	Occ. (<1)
Ni	0.14310 (8)	0.51145 (8)	0.50592 (10)	0.0744 (4)	
N1	0.0343 (6)	0.4084 (7)	0.4852 (3)	0.081 (2)	
N2	0.0723 (6)	0.5254 (6)	0.5661 (3)	0.071 (2)	
N3	0.2520 (6)	0.6232 (6)	0.5259 (3)	0.083 (3)	
N4	0.2174 (6)	0.4911 (6)	0.4466 (3)	0.079 (2)	
C1	0.0111 (8)	0.3714 (9)	0.4404 (5)	0.090 (3)	
C2	−0.0831 (10)	0.3000 (11)	0.4370 (6)	0.127 (5)	
H2A	−0.1124	0.2670	0.4099	0.152*	
C3	−0.1200 (9)	0.2909 (11)	0.4815 (5)	0.112 (4)	
H3A	−0.1801	0.2494	0.4914	0.135*	
C4	−0.0495 (8)	0.3568 (8)	0.5105 (5)	0.092 (3)	
C5	−0.0628 (7)	0.3698 (8)	0.5590 (4)	0.089 (3)	
C6	−0.0068 (8)	0.4544 (9)	0.5839 (4)	0.079 (3)	
C7	−0.0271 (9)	0.4850 (9)	0.6326 (4)	0.086 (3)	
H7A	−0.0735	0.4470	0.6532	0.104*	
C8	0.0330 (8)	0.5775 (8)	0.6420 (5)	0.084 (3)	
H8A	0.0326	0.6196	0.6701	0.101*	
C9	0.0978 (8)	0.6019 (8)	0.6027 (4)	0.078 (3)	
C10	0.1738 (8)	0.6880 (7)	0.6011 (3)	0.087 (3)	
C11	0.2458 (9)	0.6990 (9)	0.5649 (5)	0.095 (4)	
C12	0.3381 (11)	0.7722 (10)	0.5651 (5)	0.122 (5)	
H12A	0.3523	0.8327	0.5862	0.146*	
C13	0.4007 (10)	0.7378 (10)	0.5293 (5)	0.116 (5)	
H13A	0.4681	0.7678	0.5223	0.139*	
C14	0.3462 (8)	0.6468 (8)	0.5035 (6)	0.091 (3)	
C15	0.3855 (6)	0.5940 (9)	0.4632 (4)	0.088 (3)	
C16	0.3189 (9)	0.5242 (9)	0.4349 (5)	0.088 (3)	
C17	0.3435 (9)	0.4928 (9)	0.3878 (5)	0.094 (3)	
H17A	0.4081	0.5049	0.3722	0.112*	
C18	0.2570 (9)	0.4431 (10)	0.3705 (5)	0.094 (3)	
H18A	0.2487	0.4158	0.3398	0.113*	
C19	0.1805 (9)	0.4385 (9)	0.4056 (4)	0.088 (3)	
C20	0.0775 (9)	0.3858 (8)	0.4025 (3)	0.095 (4)	
S1A	−0.2681 (4)	0.3838 (5)	0.6016 (2)	0.146 (2)	0.796 (6)
C21A	−0.1537 (7)	0.3093 (9)	0.5828 (4)	0.094 (4)	0.796 (6)
C22A	−0.1604 (11)	0.1952 (12)	0.5935 (5)	0.138 (6)	0.796 (6)
C23A	−0.2587 (11)	0.1713 (12)	0.6198 (6)	0.118 (5)	0.796 (6)
H23A	−0.2741	0.0983	0.6328	0.142*	0.796 (6)
C24A	−0.3192 (13)	0.2555 (12)	0.6231 (7)	0.141 (6)	0.796 (6)
H24A	−0.3873	0.2501	0.6363	0.170*	0.796 (6)
C25A	−0.0842 (11)	0.1202 (10)	0.5797 (6)	0.104 (4)	0.796 (6)
H25A	−0.0163	0.1452	0.5919	0.156*	0.796 (6)
H25B	−0.0813	0.1185	0.5459	0.156*	0.796 (6)
H25C	−0.1005	0.0431	0.5913	0.156*	0.796 (6)
S1B	−0.0775 (13)	0.1502 (16)	0.6113 (8)	0.146 (2)	0.204 (6)
C21B	−0.1258 (15)	0.2723 (17)	0.5803 (10)	0.094 (4)	0.204 (6)
C22B	−0.2334 (17)	0.270 (2)	0.5802 (11)	0.138 (6)	0.204 (6)
C23B	−0.2729 (18)	0.168 (3)	0.6064 (17)	0.118 (5)	0.204 (6)
H23B	−0.3454	0.1533	0.6111	0.142*	0.204 (6)
C24B	−0.2020 (19)	0.103 (3)	0.6217 (17)	0.141 (6)	0.204 (6)
H24B	−0.2166	0.0339	0.6377	0.170*	0.204 (6)
C25B	−0.293 (3)	0.351 (3)	0.5566 (16)	0.104 (4)	0.204 (6)
H25D	−0.2679	0.3564	0.5247	0.156*	0.204 (6)
H25E	−0.2855	0.4259	0.5717	0.156*	0.204 (6)
H25F	−0.3666	0.3277	0.5568	0.156*	0.204 (6)
S2A	0.2984 (7)	0.7338 (6)	0.6854 (3)	0.142 (2)	0.579 (7)
C26A	0.1993 (12)	0.7618 (11)	0.6429 (4)	0.085 (4)	0.579 (7)
C27A	0.1426 (12)	0.8552 (14)	0.6570 (6)	0.115 (6)	0.579 (7)
C28A	0.1804 (16)	0.8939 (17)	0.7031 (7)	0.121 (6)	0.579 (7)
H28A	0.1452	0.9517	0.7202	0.145*	0.579 (7)
C29A	0.2619 (14)	0.8453 (14)	0.7179 (6)	0.104 (5)	0.579 (7)
H29A	0.2987	0.8695	0.7447	0.124*	0.579 (7)
C30A	0.0552 (14)	0.8893 (17)	0.6305 (7)	0.089 (5)	0.579 (7)
H30A	0.0728	0.9587	0.6129	0.133*	0.579 (7)
H30B	−0.0042	0.9053	0.6510	0.133*	0.579 (7)
H30C	0.0362	0.8274	0.6090	0.133*	0.579 (7)
S2B	0.0856 (10)	0.8896 (10)	0.6463 (5)	0.142 (2)	0.421 (7)
C26B	0.1713 (15)	0.7704 (13)	0.6416 (5)	0.085 (4)	0.421 (7)
C27B	0.2416 (14)	0.7757 (15)	0.6770 (7)	0.115 (6)	0.421 (7)
C28B	0.226 (2)	0.879 (2)	0.7063 (9)	0.121 (6)	0.421 (7)
H28B	0.2692	0.8978	0.7318	0.145*	0.421 (7)
C29B	0.1483 (17)	0.9389 (18)	0.6937 (8)	0.104 (5)	0.421 (7)
H29B	0.1270	1.0064	0.7096	0.124*	0.421 (7)
C30B	0.3154 (19)	0.6871 (19)	0.6832 (11)	0.089 (5)	0.421 (7)
H30D	0.3127	0.6345	0.6568	0.133*	0.421 (7)
H30E	0.2991	0.6448	0.7115	0.133*	0.421 (7)
H30F	0.3859	0.7203	0.6855	0.133*	0.421 (7)
S3A	0.5088 (8)	0.7604 (12)	0.4087 (6)	0.181 (4)	0.447 (8)
C31A	0.4899 (8)	0.6423 (15)	0.4470 (7)	0.101 (4)	0.447 (8)
C32A	0.5847 (14)	0.5906 (15)	0.4552 (7)	0.119 (7)	0.447 (8)
C33A	0.6712 (14)	0.652 (2)	0.4307 (11)	0.124 (6)	0.447 (8)
H33A	0.7432	0.6355	0.4355	0.149*	0.447 (8)
C34A	0.6397 (13)	0.729 (2)	0.4033 (9)	0.122 (6)	0.447 (8)
H34A	0.6843	0.7657	0.3816	0.147*	0.447 (8)
C35A	0.588 (2)	0.4925 (18)	0.4840 (9)	0.089 (5)	0.447 (8)
H35A	0.6399	0.4382	0.4723	0.133*	0.447 (8)
H35B	0.5184	0.4557	0.4842	0.133*	0.447 (8)
H35C	0.6063	0.5156	0.5155	0.133*	0.447 (8)
S3B	0.6010 (8)	0.5209 (9)	0.4721 (5)	0.181 (4)	0.553 (8)
C31B	0.4997 (7)	0.6126 (14)	0.4503 (7)	0.101 (4)	0.553 (8)
C32B	0.5424 (12)	0.7030 (14)	0.4258 (6)	0.119 (7)	0.553 (8)
C33B	0.6590 (12)	0.6905 (19)	0.4249 (10)	0.124 (6)	0.553 (8)
H33B	0.7033	0.7430	0.4090	0.149*	0.553 (8)
C34B	0.6949 (13)	0.6042 (16)	0.4469 (8)	0.122 (6)	0.553 (8)
H34B	0.7676	0.5878	0.4492	0.147*	0.553 (8)
C35B	0.4711 (15)	0.7776 (17)	0.4026 (8)	0.089 (5)	0.553 (8)
H35D	0.4266	0.7325	0.3817	0.133*	0.553 (8)
H35E	0.5105	0.8346	0.3847	0.133*	0.553 (8)
H35F	0.4269	0.8169	0.4254	0.133*	0.553 (8)
S4A	−0.0458 (4)	0.4210 (5)	0.3214 (2)	0.168 (2)	0.956 (7)
C36A	0.0449 (8)	0.3410 (9)	0.3554 (3)	0.108 (4)	0.956 (7)
C37A	0.0649 (9)	0.2366 (10)	0.3346 (4)	0.122 (4)	0.956 (7)
C38A	0.0050 (11)	0.2233 (13)	0.2904 (5)	0.141 (5)	0.956 (7)
H38A	0.0103	0.1578	0.2710	0.169*	0.956 (7)
C39A	−0.0527 (11)	0.3077 (12)	0.2819 (5)	0.143 (5)	0.956 (7)
H39A	−0.0974	0.3100	0.2556	0.172*	0.956 (7)
C40A	0.1352 (11)	0.1616 (11)	0.3512 (6)	0.135 (5)	0.956 (7)
H40A	0.1512	0.1806	0.3834	0.203*	0.956 (7)
H40B	0.1996	0.1657	0.3328	0.203*	0.956 (7)
H40C	0.1063	0.0836	0.3496	0.203*	0.956 (7)
S4B	0.119 (5)	0.139 (3)	0.3825 (12)	0.168 (2)	0.044 (7)
C36B	0.090 (5)	0.286 (2)	0.3691 (9)	0.108 (4)	0.044 (7)
C37B	0.061 (5)	0.294 (2)	0.3237 (11)	0.122 (4)	0.044 (7)
C38B	0.063 (8)	0.178 (3)	0.3009 (14)	0.141 (5)	0.044 (7)
H38B	0.0439	0.1669	0.2695	0.169*	0.044 (7)
C39B	0.091 (8)	0.098 (3)	0.3271 (16)	0.143 (5)	0.044 (7)
H39B	0.0971	0.0210	0.3170	0.172*	0.044 (7)
C40B	0.035 (8)	0.400 (4)	0.305 (2)	0.135 (5)	0.044 (7)
H40D	−0.0190	0.4370	0.3239	0.203*	0.044 (7)
H40E	0.0071	0.3888	0.2734	0.203*	0.044 (7)
H40F	0.0972	0.4486	0.3035	0.203*	0.044 (7)
C1S	0.136 (3)	0.662 (2)	0.2600 (11)	0.167 (11)	0.50
H1SA	0.1119	0.5848	0.2596	0.200*	0.50
C2S	0.0717 (19)	0.751 (3)	0.2425 (12)	0.264 (18)	0.50
H2SA	0.0043	0.7341	0.2304	0.316*	0.50
C3S	0.108 (2)	0.867 (3)	0.2431 (13)	0.31 (2)	0.50
H3SA	0.0649	0.9266	0.2314	0.371*	0.50
C4S	0.209 (3)	0.893 (2)	0.2612 (11)	0.169 (12)	0.50
H4SA	0.2330	0.9699	0.2616	0.203*	0.50
C5S	0.273 (2)	0.803 (3)	0.2786 (12)	0.251 (18)	0.50
H5SA	0.3406	0.8205	0.2907	0.301*	0.50
C6S	0.237 (2)	0.688 (3)	0.2780 (12)	0.276 (19)	0.50
H6SA	0.2801	0.6280	0.2897	0.332*	0.50

### Atomic displacement parameters (Å^2^)

**Table:**

	*U*^11^	*U*^22^	*U*^33^	*U*^12^	*U*^13^	*U*^23^
Ni	0.0721 (6)	0.0712 (6)	0.0800 (8)	−0.0072 (5)	−0.0074 (8)	−0.0046 (9)
N1	0.079 (5)	0.090 (5)	0.074 (6)	−0.014 (4)	−0.011 (4)	−0.026 (4)
N2	0.066 (4)	0.065 (4)	0.082 (6)	−0.010 (4)	−0.006 (4)	0.000 (4)
N3	0.079 (5)	0.078 (5)	0.091 (7)	−0.022 (4)	−0.004 (5)	0.006 (4)
N4	0.070 (5)	0.083 (5)	0.082 (7)	−0.005 (4)	−0.004 (5)	−0.008 (5)
C1	0.068 (6)	0.096 (7)	0.108 (10)	−0.020 (6)	0.013 (7)	−0.015 (7)
C2	0.125 (10)	0.130 (10)	0.125 (13)	−0.056 (9)	−0.016 (9)	−0.028 (9)
C3	0.090 (7)	0.143 (10)	0.103 (11)	−0.054 (7)	0.014 (7)	−0.017 (8)
C4	0.082 (6)	0.098 (6)	0.096 (9)	−0.027 (5)	0.000 (8)	−0.018 (8)
C5	0.083 (7)	0.081 (6)	0.102 (10)	−0.019 (6)	0.012 (7)	−0.011 (7)
C6	0.071 (6)	0.084 (6)	0.083 (9)	−0.004 (5)	0.000 (6)	0.001 (6)
C7	0.098 (7)	0.085 (6)	0.076 (8)	0.012 (6)	0.012 (6)	0.012 (6)
C8	0.091 (7)	0.060 (5)	0.101 (9)	−0.009 (5)	0.007 (7)	−0.009 (6)
C9	0.083 (6)	0.070 (6)	0.080 (8)	−0.004 (5)	−0.003 (6)	0.000 (6)
C10	0.086 (7)	0.078 (6)	0.098 (9)	−0.019 (6)	0.014 (6)	−0.008 (6)
C11	0.105 (8)	0.074 (6)	0.107 (11)	−0.021 (6)	−0.004 (7)	−0.017 (7)
C12	0.150 (11)	0.107 (8)	0.107 (11)	−0.069 (8)	0.033 (9)	−0.027 (8)
C13	0.111 (9)	0.124 (9)	0.113 (12)	−0.044 (8)	−0.001 (8)	−0.029 (8)
C14	0.081 (6)	0.089 (5)	0.103 (9)	−0.024 (5)	−0.009 (8)	−0.020 (9)
C15	0.076 (6)	0.100 (7)	0.087 (9)	−0.018 (6)	0.002 (6)	0.001 (7)
C16	0.084 (7)	0.081 (6)	0.100 (10)	0.006 (6)	0.004 (7)	0.001 (6)
C17	0.077 (7)	0.112 (8)	0.092 (10)	−0.003 (6)	0.001 (6)	0.002 (7)
C18	0.079 (7)	0.126 (8)	0.078 (9)	−0.012 (7)	0.010 (6)	−0.008 (7)
C19	0.101 (8)	0.093 (6)	0.069 (8)	−0.020 (6)	−0.009 (7)	−0.008 (6)
C20	0.089 (7)	0.095 (7)	0.102 (10)	−0.001 (6)	−0.008 (8)	−0.022 (7)
S1A	0.123 (3)	0.157 (3)	0.159 (4)	−0.008 (3)	0.028 (3)	−0.013 (3)
C21A	0.099 (6)	0.086 (6)	0.095 (7)	−0.008 (5)	−0.002 (5)	−0.004 (5)
C22A	0.131 (8)	0.142 (8)	0.141 (9)	−0.005 (6)	0.000 (6)	0.004 (6)
C23A	0.116 (7)	0.118 (6)	0.121 (8)	−0.030 (5)	−0.001 (6)	0.001 (6)
C24A	0.139 (8)	0.140 (8)	0.145 (8)	−0.011 (6)	0.006 (6)	−0.003 (6)
C25A	0.121 (7)	0.083 (6)	0.108 (7)	−0.016 (5)	−0.002 (6)	−0.014 (5)
S1B	0.123 (3)	0.157 (3)	0.159 (4)	−0.008 (3)	0.028 (3)	−0.013 (3)
C21B	0.099 (6)	0.086 (6)	0.095 (7)	−0.008 (5)	−0.002 (5)	−0.004 (5)
C22B	0.131 (8)	0.142 (8)	0.141 (9)	−0.005 (6)	0.000 (6)	0.004 (6)
C23B	0.116 (7)	0.118 (6)	0.121 (8)	−0.030 (5)	−0.001 (6)	0.001 (6)
C24B	0.139 (8)	0.140 (8)	0.145 (8)	−0.011 (6)	0.006 (6)	−0.003 (6)
C25B	0.121 (7)	0.083 (6)	0.108 (7)	−0.016 (5)	−0.002 (6)	−0.014 (5)
S2A	0.160 (4)	0.128 (4)	0.136 (5)	−0.004 (4)	−0.006 (4)	−0.021 (4)
C26A	0.075 (6)	0.086 (5)	0.093 (6)	−0.003 (5)	0.010 (5)	−0.012 (5)
C27A	0.120 (8)	0.114 (8)	0.112 (9)	−0.002 (6)	−0.007 (6)	−0.005 (6)
C28A	0.122 (9)	0.119 (7)	0.121 (8)	0.003 (6)	−0.005 (6)	−0.005 (6)
C29A	0.110 (7)	0.103 (7)	0.098 (8)	−0.004 (6)	0.005 (6)	−0.005 (6)
C30A	0.099 (7)	0.083 (6)	0.085 (8)	0.002 (6)	−0.015 (6)	−0.010 (6)
S2B	0.160 (4)	0.128 (4)	0.136 (5)	−0.004 (4)	−0.006 (4)	−0.021 (4)
C26B	0.075 (6)	0.086 (5)	0.093 (6)	−0.003 (5)	0.010 (5)	−0.012 (5)
C27B	0.120 (8)	0.114 (8)	0.112 (9)	−0.002 (6)	−0.007 (6)	−0.005 (6)
C28B	0.122 (9)	0.119 (7)	0.121 (8)	0.003 (6)	−0.005 (6)	−0.005 (6)
C29B	0.110 (7)	0.103 (7)	0.098 (8)	−0.004 (6)	0.005 (6)	−0.005 (6)
C30B	0.099 (7)	0.083 (6)	0.085 (8)	0.002 (6)	−0.015 (6)	−0.010 (6)
S3A	0.104 (4)	0.212 (7)	0.226 (9)	−0.015 (5)	0.032 (5)	0.073 (7)
C31A	0.098 (6)	0.099 (7)	0.107 (7)	0.001 (5)	0.003 (5)	−0.001 (6)
C32A	0.114 (8)	0.123 (8)	0.120 (9)	−0.006 (6)	0.009 (6)	−0.004 (6)
C33A	0.122 (7)	0.122 (8)	0.130 (8)	−0.003 (6)	−0.001 (6)	0.002 (6)
C34A	0.114 (8)	0.129 (8)	0.124 (9)	−0.010 (6)	0.003 (6)	0.003 (6)
C35A	0.079 (7)	0.094 (7)	0.094 (8)	−0.010 (6)	0.006 (6)	0.013 (6)
S3B	0.104 (4)	0.212 (7)	0.226 (9)	−0.015 (5)	0.032 (5)	0.073 (7)
C31B	0.098 (6)	0.099 (7)	0.107 (7)	0.001 (5)	0.003 (5)	−0.001 (6)
C32B	0.114 (8)	0.123 (8)	0.120 (9)	−0.006 (6)	0.009 (6)	−0.004 (6)
C33B	0.122 (7)	0.122 (8)	0.130 (8)	−0.003 (6)	−0.001 (6)	0.002 (6)
C34B	0.114 (8)	0.129 (8)	0.124 (9)	−0.010 (6)	0.003 (6)	0.003 (6)
C35B	0.079 (7)	0.094 (7)	0.094 (8)	−0.010 (6)	0.006 (6)	0.013 (6)
S4A	0.163 (4)	0.200 (4)	0.141 (4)	−0.030 (3)	−0.040 (3)	0.007 (3)
C36A	0.099 (6)	0.120 (6)	0.105 (7)	−0.015 (5)	−0.008 (5)	−0.002 (5)
C37A	0.130 (6)	0.128 (7)	0.109 (7)	−0.019 (6)	−0.010 (6)	−0.012 (6)
C38A	0.140 (7)	0.153 (7)	0.131 (8)	−0.011 (6)	−0.004 (6)	−0.017 (6)
C39A	0.147 (7)	0.150 (7)	0.133 (8)	−0.016 (6)	−0.008 (6)	−0.007 (6)
C40A	0.141 (7)	0.129 (6)	0.135 (8)	−0.002 (6)	0.016 (6)	−0.033 (6)
S4B	0.163 (4)	0.200 (4)	0.141 (4)	−0.030 (3)	−0.040 (3)	0.007 (3)
C36B	0.099 (6)	0.120 (6)	0.105 (7)	−0.015 (5)	−0.008 (5)	−0.002 (5)
C37B	0.130 (6)	0.128 (7)	0.109 (7)	−0.019 (6)	−0.010 (6)	−0.012 (6)
C38B	0.140 (7)	0.153 (7)	0.131 (8)	−0.011 (6)	−0.004 (6)	−0.017 (6)
C39B	0.147 (7)	0.150 (7)	0.133 (8)	−0.016 (6)	−0.008 (6)	−0.007 (6)
C40B	0.141 (7)	0.129 (6)	0.135 (8)	−0.002 (6)	0.016 (6)	−0.033 (6)
C1S	0.176 (14)	0.156 (13)	0.167 (14)	−0.006 (9)	−0.001 (9)	−0.003 (9)
C2S	0.263 (19)	0.26 (2)	0.26 (2)	0.000 (10)	−0.001 (10)	−0.002 (10)
C3S	0.31 (2)	0.31 (2)	0.31 (2)	0.007 (10)	−0.002 (10)	0.002 (10)
C4S	0.176 (14)	0.156 (13)	0.174 (15)	0.000 (9)	−0.007 (9)	−0.007 (9)
C5S	0.248 (19)	0.254 (19)	0.25 (2)	−0.002 (10)	0.000 (10)	−0.004 (10)
C6S	0.28 (2)	0.27 (2)	0.28 (2)	0.005 (10)	0.006 (10)	−0.001 (10)

### Geometric parameters (Å, º)

**Table:**

Ni—N1	1.888 (8)	C27A—C28A	1.458 (15)
Ni—N2	1.930 (9)	C28A—C29A	1.232 (17)
Ni—N4	1.936 (9)	C28A—H28A	0.9300
Ni—N3	1.948 (8)	C29A—H29A	0.9300
N1—C1	1.371 (15)	C30A—H30A	0.9600
N1—C4	1.397 (14)	C30A—H30B	0.9600
N2—C6	1.372 (12)	C30A—H30C	0.9600
N2—C9	1.391 (13)	S2B—C29B	1.654 (16)
N3—C14	1.364 (13)	S2B—C26B	1.734 (14)
N3—C11	1.404 (14)	C26B—C27B	1.334 (16)
N4—C16	1.363 (13)	C27B—C30B	1.378 (16)
N4—C19	1.386 (14)	C27B—C28B	1.454 (16)
C1—C20	1.367 (15)	C28B—C29B	1.237 (18)
C1—C2	1.433 (15)	C28B—H28B	0.9300
C2—C3	1.350 (18)	C29B—H29B	0.9300
C2—H2A	0.9300	C30B—H30D	0.9600
C3—C4	1.419 (16)	C30B—H30E	0.9600
C3—H3A	0.9300	C30B—H30F	0.9600
C4—C5	1.396 (16)	S3A—C34A	1.680 (15)
C5—C6	1.383 (14)	S3A—C31A	1.746 (14)
C5—C21B	1.488 (7)	C31A—C32A	1.342 (16)
C5—C21A	1.489 (6)	C32A—C35A	1.384 (16)
C6—C7	1.448 (16)	C32A—C33A	1.464 (16)
C7—C8	1.321 (13)	C33A—C34A	1.234 (18)
C7—H7A	0.9300	C33A—H33A	0.9300
C8—C9	1.405 (15)	C34A—H34A	0.9300
C8—H8A	0.9300	C35A—H35A	0.9600
C9—C10	1.366 (13)	C35A—H35B	0.9600
C10—C11	1.370 (15)	C35A—H35C	0.9600
C10—C26B	1.486 (7)	S3B—C34B	1.669 (15)
C10—C26A	1.488 (7)	S3B—C31B	1.755 (14)
C11—C12	1.422 (14)	C31B—C32B	1.351 (15)
C12—C13	1.339 (16)	C32B—C35B	1.396 (16)
C12—H12A	0.9300	C32B—C33B	1.463 (15)
C13—C14	1.440 (15)	C33B—C34B	1.248 (18)
C13—H13A	0.9300	C33B—H33B	0.9300
C14—C15	1.380 (16)	C34B—H34B	0.9300
C15—C16	1.404 (15)	C35B—H35D	0.9600
C15—C31B	1.488 (7)	C35B—H35E	0.9600
C15—C31A	1.488 (7)	C35B—H35F	0.9600
C16—C17	1.417 (17)	S4A—C39A	1.713 (12)
C17—C18	1.314 (14)	S4A—C36A	1.743 (10)
C17—H17A	0.9300	C36A—C37A	1.350 (12)
C18—C19	1.382 (15)	C37A—C40A	1.311 (13)
C18—H18A	0.9300	C37A—C38A	1.469 (14)
C19—C20	1.422 (14)	C38A—C39A	1.226 (14)
C20—C36A	1.487 (6)	C38A—H38A	0.9300
C20—C36B	1.488 (7)	C39A—H39A	0.9300
S1A—C24A	1.708 (13)	C40A—H40A	0.9600
S1A—C21A	1.744 (10)	C40A—H40B	0.9600
C21A—C22A	1.337 (14)	C40A—H40C	0.9600
C22A—C25A	1.337 (14)	S4B—C39B	1.674 (17)
C22A—C23A	1.461 (14)	S4B—C36B	1.751 (16)
C23A—C24A	1.225 (15)	C36B—C37B	1.343 (17)
C23A—H23A	0.9300	C37B—C40B	1.363 (18)
C24A—H24A	0.9300	C37B—C38B	1.462 (17)
C25A—H25A	0.9600	C38B—C39B	1.231 (19)
C25A—H25B	0.9600	C38B—H38B	0.9300
C25A—H25C	0.9600	C39B—H39B	0.9300
S1B—C24B	1.670 (17)	C40B—H40D	0.9600
S1B—C21B	1.752 (15)	C40B—H40E	0.9600
C21B—C22B	1.344 (17)	C40B—H40F	0.9600
C22B—C25B	1.362 (17)	C1S—C2S	1.3900
C22B—C23B	1.461 (17)	C1S—C6S	1.3900
C23B—C24B	1.230 (19)	C1S—H1SA	0.9300
C23B—H23B	0.9300	C2S—C3S	1.3900
C24B—H24B	0.9300	C2S—H2SA	0.9300
C25B—H25D	0.9600	C3S—C4S	1.3900
C25B—H25E	0.9600	C3S—H3SA	0.9300
C25B—H25F	0.9600	C4S—C5S	1.3900
S2A—C29A	1.632 (14)	C4S—H4SA	0.9300
S2A—C26A	1.758 (13)	C5S—C6S	1.3900
C26A—C27A	1.339 (15)	C5S—H5SA	0.9300
C27A—C30A	1.381 (16)	C6S—H6SA	0.9300
			
N1—Ni—N2	89.8 (3)	C26A—C27A—C30A	118.5 (14)
N1—Ni—N4	90.0 (4)	C26A—C27A—C28A	109.7 (12)
N2—Ni—N4	177.4 (3)	C30A—C27A—C28A	131.2 (15)
N1—Ni—N3	177.5 (4)	C29A—C28A—C27A	115.9 (15)
N2—Ni—N3	90.5 (3)	C29A—C28A—H28A	122.0
N4—Ni—N3	89.8 (4)	C27A—C28A—H28A	122.0
C1—N1—C4	100.8 (9)	C28A—C29A—S2A	112.8 (13)
C1—N1—Ni	129.2 (7)	C28A—C29A—H29A	123.6
C4—N1—Ni	129.9 (8)	S2A—C29A—H29A	123.6
C6—N2—C9	105.1 (9)	C27A—C30A—H30A	109.5
C6—N2—Ni	127.3 (7)	C27A—C30A—H30B	109.5
C9—N2—Ni	127.3 (6)	H30A—C30A—H30B	109.5
C14—N3—C11	107.1 (8)	C27A—C30A—H30C	109.5
C14—N3—Ni	126.5 (8)	H30A—C30A—H30C	109.5
C11—N3—Ni	126.3 (7)	H30B—C30A—H30C	109.5
C16—N4—C19	103.0 (10)	C29B—S2B—C26B	92.1 (9)
C16—N4—Ni	128.6 (8)	C27B—C26B—C10	126.7 (14)
C19—N4—Ni	128.4 (7)	C27B—C26B—S2B	108.3 (10)
C20—C1—N1	124.3 (9)	C10—C26B—S2B	124.5 (12)
C20—C1—C2	120.8 (12)	C26B—C27B—C30B	120.2 (17)
N1—C1—C2	114.3 (11)	C26B—C27B—C28B	112.1 (13)
C3—C2—C1	105.1 (12)	C30B—C27B—C28B	127.6 (18)
C3—C2—H2A	127.5	C29B—C28B—C27B	112.8 (16)
C1—C2—H2A	127.5	C29B—C28B—H28B	123.6
C2—C3—C4	106.8 (11)	C27B—C28B—H28B	123.6
C2—C3—H3A	126.6	C28B—C29B—S2B	114.7 (15)
C4—C3—H3A	126.6	C28B—C29B—H29B	122.7
C5—C4—N1	123.4 (9)	S2B—C29B—H29B	122.7
C5—C4—C3	123.6 (11)	C27B—C30B—H30D	109.5
N1—C4—C3	113.0 (12)	C27B—C30B—H30E	109.5
C6—C5—C4	121.0 (8)	H30D—C30B—H30E	109.5
C6—C5—C21B	125.5 (16)	C27B—C30B—H30F	109.5
C4—C5—C21B	112.7 (15)	H30D—C30B—H30F	109.5
C6—C5—C21A	118.5 (11)	H30E—C30B—H30F	109.5
C4—C5—C21A	119.3 (10)	C34A—S3A—C31A	91.4 (9)
N2—C6—C5	126.0 (10)	C32A—C31A—C15	123.8 (13)
N2—C6—C7	109.5 (9)	C32A—C31A—S3A	109.1 (9)
C5—C6—C7	124.4 (10)	C15—C31A—S3A	126.6 (11)
C8—C7—C6	106.6 (10)	C31A—C32A—C35A	118.7 (17)
C8—C7—H7A	126.7	C31A—C32A—C33A	110.9 (13)
C6—C7—H7A	126.7	C35A—C32A—C33A	130.5 (17)
C7—C8—C9	108.9 (11)	C34A—C33A—C32A	113.9 (16)
C7—C8—H8A	125.6	C34A—C33A—H33A	123.1
C9—C8—H8A	125.6	C32A—C33A—H33A	123.1
C10—C9—N2	125.7 (10)	C33A—C34A—S3A	113.7 (15)
C10—C9—C8	124.6 (10)	C33A—C34A—H34A	123.1
N2—C9—C8	109.7 (9)	S3A—C34A—H34A	123.1
C9—C10—C11	123.2 (9)	C32A—C35A—H35A	109.5
C9—C10—C26B	114.4 (11)	C32A—C35A—H35B	109.5
C11—C10—C26B	122.4 (11)	H35A—C35A—H35B	109.5
C9—C10—C26A	121.8 (10)	C32A—C35A—H35C	109.5
C11—C10—C26A	113.9 (10)	H35A—C35A—H35C	109.5
C10—C11—N3	124.7 (9)	H35B—C35A—H35C	109.5
C10—C11—C12	125.6 (11)	C34B—S3B—C31B	90.9 (8)
N3—C11—C12	108.6 (11)	C32B—C31B—C15	127.9 (12)
C13—C12—C11	107.4 (11)	C32B—C31B—S3B	110.5 (8)
C13—C12—H12A	126.3	C15—C31B—S3B	121.3 (10)
C11—C12—H12A	126.3	C31B—C32B—C35B	117.0 (14)
C12—C13—C14	108.7 (11)	C31B—C32B—C33B	109.1 (12)
C12—C13—H13A	125.7	C35B—C32B—C33B	133.4 (16)
C14—C13—H13A	125.7	C34B—C33B—C32B	115.1 (15)
N3—C14—C15	127.3 (8)	C34B—C33B—H33B	122.4
N3—C14—C13	108.2 (12)	C32B—C33B—H33B	122.4
C15—C14—C13	124.5 (11)	C33B—C34B—S3B	114.3 (13)
C14—C15—C16	120.7 (8)	C33B—C34B—H34B	122.9
C14—C15—C31B	118.9 (11)	S3B—C34B—H34B	122.9
C16—C15—C31B	120.5 (12)	C32B—C35B—H35D	109.5
C14—C15—C31A	114.0 (12)	C32B—C35B—H35E	109.5
C16—C15—C31A	123.5 (13)	H35D—C35B—H35E	109.5
N4—C16—C15	124.6 (11)	C32B—C35B—H35F	109.5
N4—C16—C17	111.2 (10)	H35D—C35B—H35F	109.5
C15—C16—C17	123.7 (11)	H35E—C35B—H35F	109.5
C18—C17—C16	106.4 (11)	C39A—S4A—C36A	90.1 (6)
C18—C17—H17A	126.8	C37A—C36A—C20	130.0 (10)
C16—C17—H17A	126.8	C37A—C36A—S4A	109.9 (8)
C17—C18—C19	108.4 (12)	C20—C36A—S4A	119.6 (8)
C17—C18—H18A	125.8	C40A—C37A—C36A	122.8 (11)
C19—C18—H18A	125.8	C40A—C37A—C38A	125.5 (12)
C18—C19—N4	111.0 (9)	C36A—C37A—C38A	111.6 (11)
C18—C19—C20	126.6 (11)	C39A—C38A—C37A	112.8 (13)
N4—C19—C20	122.4 (10)	C39A—C38A—H38A	123.6
C1—C20—C19	123.4 (9)	C37A—C38A—H38A	123.6
C1—C20—C36A	119.9 (10)	C38A—C39A—S4A	115.5 (11)
C19—C20—C36A	116.6 (10)	C38A—C39A—H39A	122.2
C1—C20—C36B	118 (2)	S4A—C39A—H39A	122.2
C19—C20—C36B	106 (3)	C37A—C40A—H40A	109.5
C24A—S1A—C21A	90.0 (6)	C37A—C40A—H40B	109.5
C22A—C21A—C5	126.9 (10)	H40A—C40A—H40B	109.5
C22A—C21A—S1A	110.6 (8)	C37A—C40A—H40C	109.5
C5—C21A—S1A	122.5 (8)	H40A—C40A—H40C	109.5
C25A—C22A—C21A	120.7 (12)	H40B—C40A—H40C	109.5
C25A—C22A—C23A	128.9 (13)	C39B—S4B—C36B	91.1 (11)
C21A—C22A—C23A	110.4 (11)	C37B—C36B—C20	122 (3)
C24A—C23A—C22A	114.4 (13)	C37B—C36B—S4B	109.1 (13)
C24A—C23A—H23A	122.8	C20—C36B—S4B	128 (2)
C22A—C23A—H23A	122.8	C36B—C37B—C40B	120 (2)
C23A—C24A—S1A	114.3 (12)	C36B—C37B—C38B	111.1 (15)
C23A—C24A—H24A	122.9	C40B—C37B—C38B	129 (2)
S1A—C24A—H24A	122.9	C39B—C38B—C37B	113.8 (18)
C22A—C25A—H25A	109.5	C39B—C38B—H38B	123.1
C22A—C25A—H25B	109.5	C37B—C38B—H38B	123.1
H25A—C25A—H25B	109.5	C38B—C39B—S4B	114.8 (17)
C22A—C25A—H25C	109.5	C38B—C39B—H39B	122.6
H25A—C25A—H25C	109.5	S4B—C39B—H39B	122.6
H25B—C25A—H25C	109.5	C37B—C40B—H40D	109.5
C24B—S1B—C21B	91.3 (10)	C37B—C40B—H40E	109.5
C22B—C21B—C5	123.0 (16)	H40D—C40B—H40E	109.5
C22B—C21B—S1B	109.1 (11)	C37B—C40B—H40F	109.5
C5—C21B—S1B	127.9 (14)	H40D—C40B—H40F	109.5
C21B—C22B—C25B	122 (2)	H40E—C40B—H40F	109.5
C21B—C22B—C23B	110.7 (14)	C2S—C1S—C6S	120.0
C25B—C22B—C23B	127 (2)	C2S—C1S—H1SA	120.0
C24B—C23B—C22B	114.2 (17)	C6S—C1S—H1SA	120.0
C24B—C23B—H23B	122.9	C3S—C2S—C1S	120.0
C22B—C23B—H23B	122.9	C3S—C2S—H2SA	120.0
C23B—C24B—S1B	114.6 (16)	C1S—C2S—H2SA	120.0
C23B—C24B—H24B	122.7	C2S—C3S—C4S	120.0
S1B—C24B—H24B	122.7	C2S—C3S—H3SA	120.0
C22B—C25B—H25D	109.5	C4S—C3S—H3SA	120.0
C22B—C25B—H25E	109.5	C5S—C4S—C3S	120.0
H25D—C25B—H25E	109.5	C5S—C4S—H4SA	120.0
C22B—C25B—H25F	109.5	C3S—C4S—H4SA	120.0
H25D—C25B—H25F	109.5	C4S—C5S—C6S	120.0
H25E—C25B—H25F	109.5	C4S—C5S—H5SA	120.0
C29A—S2A—C26A	92.9 (7)	C6S—C5S—H5SA	120.0
C27A—C26A—C10	125.0 (12)	C5S—C6S—C1S	120.0
C27A—C26A—S2A	108.1 (8)	C5S—C6S—H6SA	120.0
C10—C26A—S2A	126.6 (10)	C1S—C6S—H6SA	120.0
			
N2—Ni—N1—C1	165.7 (9)	S1A—C21A—C22A—C25A	175.7 (12)
N4—Ni—N1—C1	−16.8 (9)	C5—C21A—C22A—C23A	175.9 (14)
N2—Ni—N1—C4	−8.3 (8)	S1A—C21A—C22A—C23A	−3.1 (13)
N4—Ni—N1—C4	169.1 (8)	C25A—C22A—C23A—C24A	−172.6 (17)
N1—Ni—N2—C6	15.1 (8)	C21A—C22A—C23A—C24A	6 (2)
N3—Ni—N2—C6	−167.3 (8)	C22A—C23A—C24A—S1A	−6 (2)
N1—Ni—N2—C9	−171.9 (8)	C21A—S1A—C24A—C23A	3.7 (16)
N3—Ni—N2—C9	5.6 (8)	C6—C5—C21B—C22B	108 (2)
N2—Ni—N3—C14	168.2 (9)	C4—C5—C21B—C22B	−83 (3)
N4—Ni—N3—C14	−9.2 (9)	C21A—C5—C21B—C22B	30 (3)
N2—Ni—N3—C11	−15.2 (9)	C6—C5—C21B—S1B	−69 (3)
N4—Ni—N3—C11	167.4 (9)	C4—C5—C21B—S1B	101 (2)
N1—Ni—N4—C16	−168.1 (8)	C21A—C5—C21B—S1B	−147 (6)
N3—Ni—N4—C16	14.3 (8)	C24B—S1B—C21B—C22B	0.3 (14)
N1—Ni—N4—C19	11.3 (8)	C24B—S1B—C21B—C5	177 (4)
N3—Ni—N4—C19	−166.2 (8)	C5—C21B—C22B—C25B	6 (4)
C4—N1—C1—C20	−171.2 (10)	S1B—C21B—C22B—C25B	−177 (2)
Ni—N1—C1—C20	13.5 (16)	C5—C21B—C22B—C23B	−176 (3)
C4—N1—C1—C2	0.2 (12)	S1B—C21B—C22B—C23B	1.0 (18)
Ni—N1—C1—C2	−175.2 (8)	C21B—C22B—C23B—C24B	−3 (4)
C20—C1—C2—C3	171.2 (12)	C25B—C22B—C23B—C24B	175 (3)
N1—C1—C2—C3	−0.5 (15)	C22B—C23B—C24B—S1B	3 (5)
C1—C2—C3—C4	0.6 (15)	C21B—S1B—C24B—C23B	−2 (3)
C1—N1—C4—C5	−179.2 (10)	C9—C10—C26A—C27A	−80.9 (16)
Ni—N1—C4—C5	−3.9 (15)	C11—C10—C26A—C27A	110.9 (15)
C1—N1—C4—C3	0.2 (12)	C26B—C10—C26A—C27A	−19 (4)
Ni—N1—C4—C3	175.5 (8)	C9—C10—C26A—S2A	91.8 (15)
C2—C3—C4—C5	178.9 (12)	C11—C10—C26A—S2A	−76.4 (15)
C2—C3—C4—N1	−0.6 (15)	C26B—C10—C26A—S2A	153 (6)
N1—C4—C5—C6	13.7 (16)	C29A—S2A—C26A—C27A	−1.1 (10)
C3—C4—C5—C6	−165.7 (11)	C29A—S2A—C26A—C10	−174.8 (15)
N1—C4—C5—C21B	−156.2 (12)	C10—C26A—C27A—C30A	−2 (2)
C3—C4—C5—C21B	24.3 (17)	S2A—C26A—C27A—C30A	−175.9 (13)
N1—C4—C5—C21A	−178.8 (9)	C10—C26A—C27A—C28A	170.8 (17)
C3—C4—C5—C21A	1.7 (16)	S2A—C26A—C27A—C28A	−3.0 (13)
C9—N2—C6—C5	175.1 (10)	C26A—C27A—C28A—C29A	8 (2)
Ni—N2—C6—C5	−10.7 (14)	C30A—C27A—C28A—C29A	179.4 (19)
C9—N2—C6—C7	−2.2 (10)	C27A—C28A—C29A—S2A	−9 (2)
Ni—N2—C6—C7	172.0 (6)	C26A—S2A—C29A—C28A	5.7 (17)
C4—C5—C6—N2	−6.3 (16)	C9—C10—C26B—C27B	107.3 (18)
C21B—C5—C6—N2	162.3 (13)	C11—C10—C26B—C27B	−73 (2)
C21A—C5—C6—N2	−173.9 (9)	C26A—C10—C26B—C27B	−18 (4)
C4—C5—C6—C7	170.6 (10)	C9—C10—C26B—S2B	−82.1 (16)
C21B—C5—C6—C7	−20.8 (18)	C11—C10—C26B—S2B	97.2 (17)
C21A—C5—C6—C7	3.0 (15)	C26A—C10—C26B—S2B	153 (6)
N2—C6—C7—C8	4.5 (11)	C29B—S2B—C26B—C27B	0.6 (12)
C5—C6—C7—C8	−172.9 (10)	C29B—S2B—C26B—C10	−171.5 (19)
C6—C7—C8—C9	−4.8 (11)	C10—C26B—C27B—C30B	−12 (3)
C6—N2—C9—C10	179.4 (10)	S2B—C26B—C27B—C30B	176.2 (16)
Ni—N2—C9—C10	5.2 (14)	C10—C26B—C27B—C28B	170 (2)
C6—N2—C9—C8	−0.7 (10)	S2B—C26B—C27B—C28B	−1.7 (16)
Ni—N2—C9—C8	−174.9 (6)	C26B—C27B—C28B—C29B	2 (3)
C7—C8—C9—C10	−176.5 (10)	C30B—C27B—C28B—C29B	−175 (2)
C7—C8—C9—N2	3.6 (12)	C27B—C28B—C29B—S2B	−2 (3)
N2—C9—C10—C11	−9.7 (16)	C26B—S2B—C29B—C28B	1 (2)
C8—C9—C10—C11	170.4 (11)	C14—C15—C31A—C32A	100.1 (19)
N2—C9—C10—C26B	169.6 (11)	C16—C15—C31A—C32A	−95.5 (19)
C8—C9—C10—C26B	−10.3 (16)	C31B—C15—C31A—C32A	−13 (5)
N2—C9—C10—C26A	−176.8 (10)	C14—C15—C31A—S3A	−88.9 (18)
C8—C9—C10—C26A	3.3 (17)	C16—C15—C31A—S3A	75.5 (19)
C9—C10—C11—N3	−1.2 (18)	C31B—C15—C31A—S3A	158 (7)
C26B—C10—C11—N3	179.6 (12)	C34A—S3A—C31A—C32A	3.3 (12)
C26A—C10—C11—N3	166.9 (11)	C34A—S3A—C31A—C15	−169 (2)
C9—C10—C11—C12	−167.9 (11)	C15—C31A—C32A—C35A	−6 (3)
C26B—C10—C11—C12	12.9 (19)	S3A—C31A—C32A—C35A	−178.4 (16)
C26A—C10—C11—C12	0.2 (17)	C15—C31A—C32A—C33A	174 (2)
C14—N3—C11—C10	−167.2 (11)	S3A—C31A—C32A—C33A	1.8 (15)
Ni—N3—C11—C10	15.7 (16)	C31A—C32A—C33A—C34A	−8 (3)
C14—N3—C11—C12	1.5 (13)	C35A—C32A—C33A—C34A	172 (2)
Ni—N3—C11—C12	−175.6 (8)	C32A—C33A—C34A—S3A	11 (3)
C10—C11—C12—C13	165.2 (12)	C31A—S3A—C34A—C33A	−9 (2)
N3—C11—C12—C13	−3.3 (15)	C14—C15—C31B—C32B	−84.9 (19)
C11—C12—C13—C14	3.8 (16)	C16—C15—C31B—C32B	95.0 (19)
C11—N3—C14—C15	−179.5 (11)	C31A—C15—C31B—C32B	−11 (5)
Ni—N3—C14—C15	−2.4 (17)	C14—C15—C31B—S3B	87.9 (16)
C11—N3—C14—C13	0.8 (13)	C16—C15—C31B—S3B	−92.2 (16)
Ni—N3—C14—C13	177.9 (8)	C31A—C15—C31B—S3B	161 (7)
C12—C13—C14—N3	−3.0 (15)	C34B—S3B—C31B—C32B	−1.2 (11)
C12—C13—C14—C15	177.4 (12)	C34B—S3B—C31B—C15	−175.1 (18)
N3—C14—C15—C16	13.6 (18)	C15—C31B—C32B—C35B	−12 (2)
C13—C14—C15—C16	−166.8 (11)	S3B—C31B—C32B—C35B	174.3 (14)
N3—C14—C15—C31B	−166.5 (11)	C15—C31B—C32B—C33B	175 (2)
C13—C14—C15—C31B	13.1 (18)	S3B—C31B—C32B—C33B	1.7 (14)
N3—C14—C15—C31A	178.5 (12)	C31B—C32B—C33B—C34B	−2 (3)
C13—C14—C15—C31A	−1.9 (18)	C35B—C32B—C33B—C34B	−173 (2)
C19—N4—C16—C15	172.1 (10)	C32B—C33B—C34B—S3B	1 (3)
Ni—N4—C16—C15	−8.3 (15)	C31B—S3B—C34B—C33B	0 (2)
C19—N4—C16—C17	0.2 (11)	C1—C20—C36A—C37A	−92.4 (14)
Ni—N4—C16—C17	179.7 (7)	C19—C20—C36A—C37A	85.2 (14)
C14—C15—C16—N4	−8.0 (17)	C36B—C20—C36A—C37A	5 (4)
C31B—C15—C16—N4	172.1 (11)	C1—C20—C36A—S4A	79.0 (12)
C31A—C15—C16—N4	−171.4 (11)	C19—C20—C36A—S4A	−103.4 (10)
C14—C15—C16—C17	162.9 (11)	C36B—C20—C36A—S4A	177 (4)
C31B—C15—C16—C17	−17.0 (17)	C39A—S4A—C36A—C37A	0.9 (9)
C31A—C15—C16—C17	−0.4 (18)	C39A—S4A—C36A—C20	−172.1 (10)
N4—C16—C17—C18	1.3 (13)	C20—C36A—C37A—C40A	−12.0 (19)
C15—C16—C17—C18	−170.8 (10)	S4A—C36A—C37A—C40A	175.9 (11)
C16—C17—C18—C19	−2.2 (13)	C20—C36A—C37A—C38A	172.2 (12)
C17—C18—C19—N4	2.4 (14)	S4A—C36A—C37A—C38A	0.1 (11)
C17—C18—C19—C20	−176.3 (10)	C40A—C37A—C38A—C39A	−177.3 (15)
C16—N4—C19—C18	−1.5 (12)	C36A—C37A—C38A—C39A	−1.7 (17)
Ni—N4—C19—C18	178.9 (7)	C37A—C38A—C39A—S4A	2.5 (18)
C16—N4—C19—C20	177.3 (9)	C36A—S4A—C39A—C38A	−2.1 (14)
Ni—N4—C19—C20	−2.3 (14)	C1—C20—C36B—C37B	118 (4)
N1—C1—C20—C19	1.8 (17)	C19—C20—C36B—C37B	−100 (4)
C2—C1—C20—C19	−169.0 (11)	C36A—C20—C36B—C37B	14 (3)
N1—C1—C20—C36A	179.2 (10)	C1—C20—C36B—S4B	−50 (6)
C2—C1—C20—C36A	8.4 (16)	C19—C20—C36B—S4B	92 (5)
N1—C1—C20—C36B	138 (2)	C36A—C20—C36B—S4B	−154 (7)
C2—C1—C20—C36B	−33 (3)	C39B—S4B—C36B—C37B	0.1 (16)
C18—C19—C20—C1	171.4 (12)	C39B—S4B—C36B—C20	169 (7)
N4—C19—C20—C1	−7.2 (16)	C20—C36B—C37B—C40B	11 (7)
C18—C19—C20—C36A	−6.1 (16)	S4B—C36B—C37B—C40B	−179 (2)
N4—C19—C20—C36A	175.3 (9)	C20—C36B—C37B—C38B	−170 (6)
C18—C19—C20—C36B	31 (2)	S4B—C36B—C37B—C38B	0.3 (19)
N4—C19—C20—C36B	−147.4 (16)	C36B—C37B—C38B—C39B	−1 (4)
C6—C5—C21A—C22A	−115.9 (13)	C40B—C37B—C38B—C39B	179 (4)
C4—C5—C21A—C22A	76.3 (16)	C37B—C38B—C39B—S4B	1 (5)
C21B—C5—C21A—C22A	−1 (4)	C36B—S4B—C39B—C38B	−1 (4)
C6—C5—C21A—S1A	63.0 (14)	C6S—C1S—C2S—C3S	0.0
C4—C5—C21A—S1A	−104.8 (11)	C1S—C2S—C3S—C4S	0.0
C21B—C5—C21A—S1A	178 (4)	C2S—C3S—C4S—C5S	0.0
C24A—S1A—C21A—C22A	0.1 (10)	C3S—C4S—C5S—C6S	0.0
C24A—S1A—C21A—C5	−179.0 (12)	C4S—C5S—C6S—C1S	0.0
C5—C21A—C22A—C25A	−5 (2)	C2S—C1S—C6S—C5S	0.0

### Hydrogen-bond geometry (Å, º)

**Table:**

*D*—H···*A*	*D*—H	H···*A*	*D*···*A*	*D*—H···*A*
C30*A*—H30*A*···S1*A*^i^	0.96	2.70	3.50 (2)	141

Symmetry code: (i) *x*+1/2, −*y*+3/2, *z*.

## References

[bb1] Agilent (2012). *CrysAlis PRO.* Agilent Technologies, Yarnton, England.

[bb2] Boyle, N. M., Rochford, J. & Pryce, M. T. (2010). *Coord. Chem. Rev.* **254**, 77–102.

[bb3] Chen, W., Akhighe, J., Brückner, C., Li, C. M. & Lei, Y. (2010). *J. Phys. Chem. C*, **114**, 8633–8638.

[bb4] Clark, R. C. & Reid, J. S. (1995). *Acta Cryst.* A**51**, 887–897.

[bb5] Flack, H. D. (1983). *Acta Cryst.* A**39**, 876–881.

[bb6] Friedlein, R., von Kieseritzky, F., Braun, S., Linde, C., Osikowicz, W., Hellberg, J. & Salaneck, W. R. (2005). *Chem. Commun.* pp. 1974–1976. 10.1039/b418866g 15834476

[bb7] Prasath, R., Bhavana, P., Ng, S. W. & Tiekink, E. R. T. (2012*a*). *Acta Cryst.* E**68**, m471–m472. 10.1107/S1600536812011671 PMC3343861 22589835

[bb8] Prasath, R., Butcher, R. J. & Bhavana, P. (2012*b*). *Spectrochim. Acta Part A*, **87**, 258–264. 10.1016/j.saa.2011.11.049 22192414

[bb9] Purushothaman, B., Varghese, B. & Bhyrappa, P. (2001). *Acta Cryst.* C**57**, 252–253. 10.1107/s0108270100018552 11250566

[bb10] Rochford, J., Botchway, S., McGarvey, J. J., Rooney, A. D. & Pryce, M. T. (2008). *J. Phys. Chem. A*, **112**, 11611–11618. 10.1021/jp805809p 18956854

[bb11] Sheldrick, G. M. (2008). *Acta Cryst.* A**64**, 112–122. 10.1107/S0108767307043930 18156677

[bb12] Song, Y., Haddad, R. E., Jia, S.-L., Hok, S., Olmstead, M. M., Nurco, D. J., Schore, N. E., Zhang, J., Ma, J.-G., Smith, K. M., Gazeau, S., Pecaut, J., Marchon, J.-C., Medforth, C. J. & Shelnutt, J. A. (2005). *J. Am. Chem. Soc.* **127**, 1179–1192. 10.1021/ja045309n 15669857

[bb13] Sun, X., Zhang, J. & He, B. (2005). *J. Photochem. Photobiol. Chem.* **172**, 283–288.

[bb14] Wallin, S., Hammarström, L., Blart, E. & Odobel, F. (2006). *Photochem. Photobiol. Sci.* **5**, 828–834. 10.1039/b601092j 17047835

